# Expertise Across Disciplines: Establishing Common Ground in Interdisciplinary Disaster Research Teams

**DOI:** 10.1111/risa.13407

**Published:** 2019-09-23

**Authors:** Jonathan M. Gilligan

**Keywords:** Collaborative research, disaster, expertise, interdisciplinary, tacit knowledge

## Abstract

Hazards and disasters arise from interactions between environmental and social processes, so interdisciplinary research is crucial in understanding and effectively managing them. Despite support and encouragement from funding agencies, universities, and journals and growing interest from researchers, interdisciplinary disaster research teams face significant obstacles, such as the difficulty of establishing effective communication and understanding across disciplines. Better understanding of interdisciplinary teamwork can also have important practical benefits for operational disaster planning and response.

Social studies of science distinguish different kinds of expertise and different modes of communication. Understanding these differences can help interdisciplinary research teams communicate more clearly and work together more effectively. The primary role of a researcher is in *contributory expertise* (the ability to make original contributions to a discipline); but *interactional expertise* in other disciplines (the ability to understand their literature and communicate with their practitioners) can play an important role in interdisciplinary collaborations. Developing interactional expertise requires time and effort, which can be challenging for a busy researcher, and also requires a foundation of trust and communication among team members. Three distinct aspects of communication play important roles in effective interdisciplinary communication: *dialects*, *metaphors*, and *articulation*. There are different ways to develop interactional expertise and effective communication, so researchers can pursue approaches that suit their circumstances. It will be important for future research on interdisciplinary disaster research to identify best practices for building trust, facilitating communication, and developing interactional expertise.

## 1 INTRODUCTION

Disasters occur at intersections between human society and processes in natural and built environments. Disaster research has long recognized the importance of interdisciplinary methods that connect social sciences, natural sciences, and engineering. Gilbert White famously observed in 1942 that “floods are ‘acts of God,’ but flood losses are largely acts of man” (White, 1942). In 1971, an expert report to the California Legislature emphasized that earthquake hazards encompassed “engineering, architecture, geology, government, and sociology” (Special Subcommittee on the San Fernando Earthquake Study, 1971, p. v). However, prominent reviews warn that in practice, disaster research and planning continue to suffer from poor engagement and communication between different disciplines and that these failures contributed to avoidable catastrophes, such as Hurricane Katrina (Fischhoff, 2006; McPhillips et al., 2018; National Research Council [NRC], 2006; White & Haas, 1975).

In recent years, the growth of federal funding lines, university initiatives, and journals specifically targeting interdisciplinary research have lowered institutional barriers. However, despite encouragement by institutions and enthusiasm from researchers, failures of implementation frequently obstruct effective disaster research (NRC, 2006). Such problems affect many kinds of interdisciplinary activities, but they are especially important for disaster research, where failure to integrate and communicate across disciplines can be a matter of life and death (Fischhoff, 2006; NRC, 2006).

Research on interdisciplinary medical research has spawned a vibrant *science of team science* (Bozeman & Youtie, 2017; Syme, 2008), but until recently there has been little systematic research on identifying best practices for establishing common intellectual ground within teams (NRC, 2015) and little of that research addresses disaster research. One review notes that “much of the knowledge that surrounds interdisciplinary research capacity‐building is tacit, with practitioners often ‘learning by doing' ” and good research practices remain largely “a matter of instinct” (Lyall & Meagher, 2012).

There is also a need for better research on teamwork in interagency disaster response, where many challenges around developing trust and effective communication are similar to those research teams face (Power, 2018). Thus, better understanding of interdisciplinary disaster research may also benefit disaster response.

Important factors for success in interdisciplinary disaster research include individual cognitive aspects, such as investigators’ understanding of one another's disciplines, and collective aspects, such as communication across disciplines and development of a “shared mental model” of the research project (Hardy, 2018; NRC, 2006). Shared understandings of different disciplines' perspectives and effective communication across disciplines provide necessary common ground for jointly asking and answering questions across disciplinary boundaries. Investigators must also be able to trust their colleagues both to show good faith and respect and also to conduct their research competently and rigorously.

Achieving such trust, shared understandings, and communicative competence requires more than casual familiarity with other disciplines; it requires detailed knowledge of the technical language, conceptual frameworks, and methods of different disciplines. Poteete, Janssen, and Ostrom (2010) emphasize that “researchers often need considerable contextual knowledge even to recognize the phenomenon of interest.” Acquiring this knowledge can seem daunting. It is difficult enough to master one discipline sufficiently to conduct original research; mastering two or more at such a level may not be realistic.

Many other factors, such as funding and institutional reward structures, affect the success of interdisciplinary research projects; but I focus here on developing understanding and effective communication because those are things a team can control. This Perspective draws both from the literature on interdisciplinarity and expertise and from my experiences in interdisciplinary collaborations among sociologists, social psychologists, political scientists, engineers, and environmental scientists investigating vulnerabilities, impacts, and adaptations to droughts, floods, tropical cyclones, and other environmental hazards. I cite examples that are largely anecdotal and it will be important for future research to move beyond anecdote with formal evaluation of interdisciplinary collaborations that will allow systematic comparisons and meta‐analysis.

### 1.1 Different Kinds of Expertise and Communication

A cognitive taxonomy of expertise developed by Collins and Evans (2008) (Table I) is helpful for addressing the tensions between acquiring deep disciplinary expertise and broad interdisciplinary expertise. One important feature that sets specialized expertise apart from popular understanding is *tacit knowledge*: understanding that cannot be reduced to a set of literal rules. Within specialized expertise, Collins and Evans distinguish between *interactional expertise* and *contributory expertise*. *Interactional expertise* in a discipline allows one to have productive conversations with other experts and to read the research literature with a solid understanding, but does not imply the ability to perform original research and advance the field. The latter skills constitute *contributory expertise*.

**Table I:** Cognitive and Linguistic Concepts in Interdisciplinary Research

Category	Concept	Description
Individual expertise	Contributory expertise	Deep mastery and ability to contribute new knowledge or methods to a discipline.
	Interactional expertise	Ability to understand a discipline's literature and converse at a high level with contributory experts.
	Tacit knowledge	Disciplinary knowledge that is not stated explicitly, but learned through experience and interactions with experts.
Group communication	Dialect	Technical vocabulary and jargon of a discipline.
	Metaphor	Verbal and graphical analogies that reflect conceptual structures of a discipline.
	Articulation	Using dialect and metaphor to express ideas in one or more disciplines.

*Note*: Cognitive aspects of individual expertise are described by Collins and Evans (2008) and linguistic aspects of interdisciplinary communication are described by Bracken and Oughton (2006).

Tacit knowledge contributes importantly to the ability to judge the quality of work in a discipline and acquiring this knowledge generally requires extensive engagement with contributory experts and drawing inferences from that experience about disciplinary judgment and practice.

Linguistic analyses of communication within interdisciplinary teams offer useful insights to facilitate understanding and effective communication across disciplines. Bracken and Oughton (2006) identify three key concepts for effective communication (Table I): *dialects*, *metaphors*, and *articulation*.

A first step in establishing a shared interdisciplinary language is to understand how different disciplines develop their own *dialects*, in which words acquire specialized meanings so the same word, such as “dynamics” or “mapping,” may mean different things to different researchers. *Metaphors* are used to make conceptual connections across disciplines, which can be important for developing novel interdisciplinary ideas and approaches. However, not all similarities and possible connections are meaningful or useful. *Articulation* provides a criterion for assessing the potential for interdisciplinary synthesis: connections that can be articulated coherently have potential and those that cannot may represent blind alleys.

## 2 DEVELOPING COMMON GROUND ACROSS DISCIPLINES

Collins and Evans's cognitive taxonomy conceives of different layers of understanding by individuals, whereas Bracken and Oughton's linguistic taxonomy describes different modes of communication in a team; but the two taxonomies have common elements.

At one level is information: a collection of technical vocabulary, facts, and rules, which can be obtained by reading books and journals. This information is necessary, but not sufficient, for understanding meaning or judging quality. Someone possessing only textbook knowledge of a field would be hard pressed to judge whether a research paper is substantive, trivial, or badly flawed. Such judgment rests not only on explicit knowledge, which can be spelled out, but also on *tacit knowledge*, which is inferred from experience and interaction with experts (e.g., studying a language with a fluent speaker or working closely with a dissertation adviser). Tacit knowledge contributes both to understanding other disciplines' languages and to developing the judgment that sets experts apart from well‐read laypeople.

Developing tacit knowledge through interaction with experts is also important for effective communication: *dialects* (technical vocabulary) often use familiar words in unfamiliar ways and novices often misunderstand conversations between experts by misinterpreting words that may have a colloquial meaning and perhaps also a technical meaning in the listeners' home discipline, but which possess distinct meanings in the speakers' discipline that are not obvious to an outsider (Bracken & Oughton, 2006; Monteiro & Keating, 2009).

Interactional expertise goes deeper than vocabulary and *metaphor* provides important modes of understanding and expressing the conceptual structure of a discipline. Within a discipline, metaphors and analogies are widely used to express complex ideas efficiently, such as representing stability and instability of complex systems in terms of balls rolling over surfaces with hills and valleys. Misunderstanding specialized uses of metaphor is a common source of misunderstanding in cross‐disciplinary communication (Bracken & Oughton, 2006; Monteiro & Keating, 2009).

Learning to understand and communicate with other disciplines imposes an opportunity cost of foregone productivity in one's home discipline, and this can be a significant barrier to interdisciplinarity (NRC, 2006; Poteete et al., 2010). However, this barrier may not be as daunting as it first seems: Acquiring contributory expertise in many disciplines is neither necessary nor realistic. Interactional expertise is easier to acquire and will facilitate effective collaboration and synthesis across disciplines.

Qualitative research has identified several common pathways that researchers follow in developing interactional expertise: independent reading combined with interdisciplinary conversations; participating in regular interdisciplinary meetings or seminars; joining interdisciplinary research projects; and team‐teaching interdisciplinary courses (Lattuca, 2001). A common theme across many studies is the importance of trust and mutual respect (Bracken & Oughton, 2006; Harris & Lyon, 2013; Monteiro & Keating, 2009). As interdisciplinary teams learn to work together, members frequently report feeling vulnerable and they are more willing to participate fully when they trust their colleagues to be respectful. Ganapati and Mostafavi (2018) have suggested that cultivating metacognition may help by promoting self‐awareness about barriers to understanding and communicating across disciplines and Hardy (2018) discusses established tools for developing metacognitive awareness around shared interdisciplinary understanding.

### 2.1 Relevance to Disaster Research

Failures in anticipating and planning for disasters often arise when specialists in certain disciplines are ignorant of phenomena well known to other disciplines (Fischhoff, 2006). Different disciplines have different ways of defining and characterizing extreme events, but most publications consider them only from limited disciplinary perspectives (McPhillips et al., 2018). This leads to conflicting, confusing, and ambiguous definitions, which can make it difficult to apply published results operationally in disaster planning.

Deadly failures in the evacuation from Hurricane Rita might have been avoided if planners had consulted behavioral scientists and empirically tested their messages to the public (Fischhoff, 2006). Even where researchers and planners know the value of experts in other disciplines, they may not realize the importance of working with them early in a project (Fischhoff, 2006). Conversely, successful integrations of geoscience with social science are making earthquake early‐warning systems more effective (Allen, Cochran, Huggins, Miles, & Otegui, 2018).

Interactional expertise and effective communication across disciplines can help disaster researchers understand what other disciplines can offer, when to bring them in, and how to work effectively with them to produce useful knowledge and to communicate results effectively to planners and the public.

Different approaches to successfully develop interdisciplinary understandings have been described in the literature, but neither in interdisciplinary disaster research nor in interdisciplinary research, more broadly, there is consensus on best practices or systematic understanding of strengths and weaknesses of different approaches (NRC, 2015).

### 2.2 Developing Interdisciplinarity Within a Project

#### 2.2.1 Intensive Focused Interactions

Focused interactions across disciplines can occur through interdisciplinary teaching, research, and interaction with colleagues during sabbatical leaves (Lattuca, 2001), and a number of successful disaster research projects have used this approach. Because funded projects are central to disaster research, I will focus on intensive team building in the course of research, but much of this can also apply to teaching and sabbatical study.

Some research projects schedule time at the beginning of a project for intensive team building to develop effective communication and shared mental models of research questions. Bracken and Oughton (2006) describe an interdisciplinary project on coupled human‐natural systems that began by developing a common language. O'Connor, Rice, Peters, and Veryzer (2003) describe regular team meetings over the course of several months at the beginning of a project to build common ground. Lanier et al. (2018) did not front‐load the team building so heavily for a large interdisciplinary project on hazards associated with sea‐level rise, but hired a professional facilitator who coordinated frequent meetings (in‐person and virtual). The facilitator tracked participation and collected feedback from participants to assess and improve the effectiveness of its process.

One disaster research project that I participated in studied cyclones, floods, and other hazards in Bangladesh: a team of 12 senior investigators and 17 junior researchers from engineering, natural sciences, and social sciences began with a week‐long intensive workshop, followed by 10 months of working together in smaller interdisciplinary groups to develop shared language, trust, and interactional expertise while designing and planning field research.

In these discussions, participants realized how differently their disciplines used terminology and thought about methods, evidence, and inference. Natural scientists and engineers tended to assume that the correct way to assess vulnerability was with the standard risk triplet (What can go wrong? How likely is it to go wrong? What are the consequences?) (Kaplan & Garrick, 1981), and that the obvious way to build resilience was to reduce the expected or worst‐case loss. In contrast, social scientists were acutely aware that *vulnerability* and *resilience* were politically loaded and contested terms (Lazrus, Morrow, Morss, & Lazo, 2012; MacKinnon & Derickson, 2013). This process produced a shared integrated conceptual framework that facilitated effective communication and collaboration (Ackerly, Anam, & Gilligan, 2015; Ackerly, Anam, Gilligan, & Goodbred, 2017).

#### 2.2.2 Collaborative Fieldwork

Collaborative fieldwork can be very effective at facilitating interdisciplinary understanding and communication. Bracken and Oughton (2006) report that discussions among team members during fieldwork contributed significantly in building common ground across disciplines. Poteete et al. (2010) used field observations in conjunction with *grounded‐theory* (iteratively using insights from observations to refine research questions and conceptual frameworks) to study socioecological systems. When it was not feasible for all team members to go into the field together, discussing experiences in the field with those who stayed behind contributed to new insights and new research questions for subsequent field seasons. O'Connor et al. (2003) describe a similar approach but note that there are significant tradeoffs between working closely together and separating to collect data more efficiently.

Ackerly et al. (2015, 2017) applied similar methods to disaster research by developing a “village transect walk” method for studying vulnerability and resilience to hazards in rural communities. Researchers would alternate between conducting their own disciplinary studies and shadowing one another to learn how their colleagues worked. Similar to *grounded‐theory* methods, investigators would reflect on how insights and observations from each other's disciplines might change the way they thought about their own research questions.

Working closely together also helped the researchers recognize when something they observed might be useful to another team member. This process proved effective for studying communities that had been catastrophically flooded by a tropical cyclone because geologists and engineers could quickly investigate sites of interest that social scientists identified through interviews and focus‐group discussions; and social scientists could ask about local perceptions and memories of features the geologists and engineers observed. These interactions allowed the team to combine disciplinary methods to understand how a large flood control project initiated in the 1950s had backfired and made communities more vulnerable to floods and severe storms (Auerbach et al., 2015; Wilson et al., 2017).

### 2.3 Gradual Approach to Interdisciplinarity

A slower and more gradual approach to interdisciplinary capacity building, outside of research projects, can offer researchers a more flexible and less demanding pathway. Interdisciplinary centers play many roles (Sá, 2008), one of which can be to host regular meetings to promote understanding, trust, and collaboration (Lattuca, 2001; Harris & Lyon, 2013). Even busy researchers can usually spare an hour to attend a weekly or monthly meeting and over time they can learn how colleagues in other disciplines think and how to communicate with them. Such centers can also train future interdisciplinary researchers by hosting postdoctoral fellows to work with investigators from multiple disciplines, thus developing strengths in collaborative research that will make them competitive for research careers and prepare them to hit the ground running. The Vanderbilt Institute for Energy and Environment at Vanderbilt University is one example of a center that has successfully developed a number of interdisciplinary disaster and hazards projects through the methods described above. Future research to compare approaches and results across interdisciplinary centers will be useful for identifying best practices.

Gradual approaches have additional advantages because when collaborators share awareness and engagement across disciplines well before developing proposals, they are better able to develop research designs centered on interdisciplinary questions and insights (Hardy, 2018). Gradual approaches may also be particularly well suited to disaster research: when a severe event strikes, researchers from different disciplines who may not have collaborated previously, but who have established relationships may be able to quickly form an investigative team and develop innovative and relevant projects that build on solid foundations of trust, communication, and shared mental models of hazards and disasters.

Institutional support for different kinds of interdisciplinary engagement outside of funded projects—both gradual, such as through interdisciplinary centers, and intensive, such as through team‐teaching—can be important both in making them possible and in conveying to faculty that their participation will be valued (Lattuca, 2001).

## 3 CONCLUSIONS

Many areas of research can benefit from interdisciplinary approaches, but interdisciplinarity is especially important for hazards and disaster research because failure to make connections across disciplines can lead to deadly errors in risk assessment, hazard mitigation, and disaster planning. Better understanding of interdisciplinary disaster research may also benefit operational emergency response, which faces similar challenges (Power, 2018).

Effective interdisciplinary disaster research requires a foundation of shared language and interactional expertise, and developing these relies in turn on trust and respect within a team. Researchers can pursue this common ground through different approaches, such as integrating foundation‐building into research projects or sustained low‐level interactions.

The variety of paths to successful interdisciplinary collaboration demonstrates that those who are interested can find an approach that suits their preferences and constraints. However, there is a need for systematic comparative research on team science in disaster research that would move beyond anecdotes of success and failure toward identifying general principles and best practices.
